# Standardised activities in wheelchair rugby, comparison between athletes with coordination impairment and athletes with other impairments

**DOI:** 10.3389/fspor.2024.1519232

**Published:** 2025-01-14

**Authors:** Viola C. Altmann, Mariska Janssen, Johanna L. J. de Wit, Rienk M. A. van der Slikke

**Affiliations:** ^1^ Klimmendaal Rehabilitation Specialists, Arnhem, Netherlands; ^2^ World Wheelchair Rugby, Etoy, Switzerland; ^3^ Peter Harrison Centre for Disability Sport, School of Sport, Exercise, and Health Sciences, Loughborough University, Loughborough, United Kingdom; ^4^ Donders Institute for Brain, Cognition and Behaviour, Radboud University, Nijmegen, Netherlands; ^5^ Centre of Expertise Health Innovation, The Hague University of Applied Sciences, The Hague, Netherlands; ^6^ Department of Biomechanical Engineering, Delft University of Technology, Delft, Netherlands

**Keywords:** ball activities, cerebral palsy, classification, coordination impairment, paralympics, wheelchair activities, wheelchair rugby, wheelchair sport

## Abstract

**Introduction:**

To determine if athletes with coordination impairment (CI) can continue playing wheelchair rugby (WR), while an evidence-based classification system, including impairment tests for CI is not yet available. This is a defensible practise if they show similar activity limitations as athletes with other eligible impairment types (OI) within the same sports class.

**Methods:**

Standardised activities were measured in 58 elite WR athletes; 14 with CI and 44 with OI. Wheelchair activities consisted of 20-meter sprint, 12-meter sprint with full stop, intermittent sprint (3-meter sprint, stop, 3-meter sprint, stop, 6-meter sprint with full stop), sprint-curve-slalom-curve, turn on the spot 180°, turn on the spot 90°, stop, turn 90°in the same direction, X-test (short circuit with sharp turns) without the ball. Ball activities consisted of maximal throwing distance, precision throwing short (25% of maximum throw) and long (75% of maximal throw) distance and X-test with the ball (pick-up the ball and dribble whilst pushing). Descriptive statistics were used and Spearman’s Rank correlation was assessed for athletes with CI and OI for each outcome measure. Differences between athletes with CI and OI were assessed using a Mann-Whitney U test.

**Results:**

Most activities showed a high correlation with the athlete class in both athletes with CI and athletes with OI. Furthermore, outcome measures of athletes with CI overlapped with athletes with OI in the same sports class for all activities. There was a trend for worse performance in athletes with CI in turn on the spot 90°, stop, turn 90°in the same direction, the short distance one handed precision throw (*P* 0.11)and in the X-test with the ball (*P* 0.10).

**Discussion:**

Despite the current lack of evidence based impairment tests for CI, it is a defensible practise to not exclude athletes with CI from WR with the current classification system. The trends for differences in performance that were found can support athletes and coaches in optimising performance of athletes with CI.

## 1 Introduction

The Paralympic Games are one of the biggest sport events worldwide, and one in which athletes with impairments have the ability to compete against each other (1). To ascertain that the best athletes win, and not simply the least impaired ones, included sports require classification to minimise the impact that impairments have on competition outcomes (2). The principal components of classification are the assessment of the impairment, and the assessment of standardised sport specific activities off court and activities on court to determine the impact of the impairment on these activities (3). In team sports, athletes with different impairment types and severities compete in one team. This puts an extra challenge on classification, because within a certain sports class, it needs to be determined if all eligible impairment types for the particular team sport have the same impact on the ability to execute sport specific activities (2–4).

Wheelchair rugby (WR) is a team sport in which proficiency is determined by wheelchair (picking, blocking and screening) and ball (ball handling, catching and passing) activities (5). Athletes are allocated a sports class depending on the severity of their impairments ranging from 0.5, 1.0 and 1.5 (low point) 2.0 and 2.5 (mid point) to 3.0 and 3.5 (high point). The sports class is made up of the average score of both arms (0.5 most severe impairment −3.5 least severe impairment) with the score for the trunk (0 most severe impairment −1.5 least severe impairment) added to derive at the sports class. At any given time, the maximum sum score of four athletes on court must be 8.0 or less (5, 6). WR was originally developed for people with tetraplegia caused by spinal cord injury. Hence, the classification system of WR is mainly based on strength impairment with objective impairment tests with manual muscle testing for all key muscles in each arm resulting in an arm score based on muscle strength profiles (6–8). However, athletes with other neuromusculoskeletal impairment types are also eligible to play the sport. In the past years, the number of participants with coordination impairment (CI) of the upper extremities due to other underlying health conditions such as cerebral palsy (CP) is on the rise. But their number is still limited, with only 5% of all athletes with an international classification having CI compared to 75% having strength impairment in 2023 (8). Contrary to the principles of classification, no objective, validated measures of the severity of arm coordination impairment are incorporated in the WR classification system (6). Instead, athletes with CI are assessed for minimum eligibility and the sports class is determined based on observation of activities that are not instrumented or standardised, and expert opinion. Descriptions of activities that determine eligibility and the sports class are described in the World Wheelchair Rugby classification rules (6). Existing tests for muscle tone (i.e., Ashworth Scale) or ataxia that are used in other sports are not routinely used. Classifiers have reported that they find classifying athletes with CI challenging, because their activity limitations seem to differ from activity limitations of athletes with other impairment types.

Any invalid classification is perceived as a threat to the Paralympic sports as it can lead to diminished success and discouragement of participation (3, 4). If a classification system does not succeed in grouping athletes with a similar impact of impairment on performance, the achievements of individual athletes can be questioned, and the sport as a whole can become less attractive for participants and spectators. Therefore, the International Paralympic Committee (IPC) published the IPC Classification Code in 2007, (revised in 2015 and 2024), which gave recognition to the need for transparent and evidence-based classification systems (2, 3). Although this was an important step for further development in the Paralympic sports, no Paralympic sport has a fully evidence-based classification system yet (9). In the classification code, CI (hypertonia, ataxia and athetosis) is one of the eligible impairment types. Coordination is defined in the IPC Classification Code as the ability to voluntarily produce skilled movement fluidly, rapidly, and accurately (10). World Wheelchair Rugby is working towards evidence-based classification, including validated tests to measure the presence and the severity of CI (11, 12). But this development takes time. At this moment objective, reliable tests have been developed, that assist in determining Minimum Impairment in CI. But additional assessment for profiles for arm scores based on the association between the outcome of these impairment tests and the performance of activities still needs to be done. Meanwhile, athletes with CP and other underlying health conditions that lead to CI increasingly participate. If the classification of these athletes that is done at this moment is seriously erroneous, their participation may have to be interrupted until evidence-based classification is developed. However, if there are no substantial differences between athletes with CI and OI, it is defensible that athletes with CI continue playing WR, while an evidence-based classification system is not yet available.

Therefore, the aim of this research is to assess if athletes with CI in WR show similar activity limitations as athletes with other eligible impairment types within the same sports class. To assess activities, existing instrumented, standardised, objective tests for wheelchair activities were used (13). However, standardised tests for ball activities that determine performance in wheelchair rugby were scarcely described in the literature. Therefore, ball activities were assessed using a newly developed test battery based on a literature review about ball activities in wheelchair sports (14).

## 2 Methods

### 2.1 Participants

In total 58 WR athletes participated in this study. Athletes were included if they played wheelchair rugby at an international level. This means they were all eligible and classified according to the rules of World Wheelchair Rugby (6). Athletes were excluded if there was any injury in their arms that could interfere with their performance. No athletes were excluded based on this criterion, because most likely, such injuries would also have prevented them from attending the tournament. It was determined if athletes had CI or another impairment type based on their classification data (8), which showed details about their underlying health condition leading to their eligible impairment type. Table 1 shows an overview of the participant characteristics. All participants play WR at an international level and were recruited at one of three international tournaments; Wheelchair Rugby World Championship 2022 (21 athletes), Musholm Cup 2023 (14 athletes) and Amsterdam Quad Rugby Tournament 2023 (23 athletes). All participants signed an informed consent before participation in this study and they signed consent to use their classification data from the classification database. The study was approved by the ethical committee of the Delft University of Technology (v1895_2022).

**Table 1:** Participant characteristics.

Characteristics	Coordination impairment (CI)	Other impairment (OI)	Total
Number of participants [*N* (%)]	14 (24.1)	44 (75.9)	58
Age (years) [Median (range)]	26 (20–34)	34 (16–47)	32 (16–47)
Gender [*N* (%)]			
Male	12 (85.7)	41 (93.2)	53 (91.4)
Female	2 (14.3)	3 (6.8)	5 (8.6)
Sports class [*N* (%)]			
Sports class 0.5	0 (0.0)	5 (11.4)	5 (8.6)
Sports class 1.0	3 (21.4)	7 (15.9)	10 (17.2)
Sports class 1.5	2 (14.3)	4 (9.1)	6 (10.3)
Sports class 2.0	3 (21.4)	7 (15.9)	10 (17.2)
Sports class 2.5	2 (14.3)	8 (18.2)	10 (17.2)
Sports class 3.0	2 (14.3)	9 (20.5)	11 (19.0)
Sports class 3.5	2 (14.3)	4 (9.1)	6 (10.3)
Median (range)	2 (1–3.5)	2 (0.5–3.5)	2 (0.5–3.5)
Hand dominance [*N* (%)]			
Right	10 (71.4)	34 (77.3)	44 (75.9)
Left	4 (28.6)	10 (22.7)	14 (24.1)

The experience of athletes in WR ranged from 6 to 18 years. Of the athletes with CI, 13 had CP, and 1 had a complex form of hereditary spastic paraplegia. The majority (32) of athletes without CI had strength impairment caused by spinal cord injury or neuromuscular disease (Charcot-Marie-Tooth polyneuropathy and muscular dystrophy). Four athletes had range impairment (Arthrogryposis Multiplex Congenital and very rare syndromes) and 8 athletes had limb length deficiencies. All athletes with CI had a score ≥ 0.5 for trunk. Athletes without CI had trunk over the full range (0–1.5). Arm scores were over the full range (0.5–3.5) for both groups.

### 2.2 Procedures

Measurements took place during the WR tournaments. If athletes agreed to participate, first athlete characteristics and information about equipment were collected. After that, the participants performed standardised wheelchair mobility performance tests and standardised ball test in a random order. All athletes were asked to provide an Fatigue Numerical Rating Scale (NRS) score before and after the tests. NRS scores ≤ 5 were considered low (15). Athletes did not perform a standardised warm-up as they either had a warm up session with their team, or they were tested after a game.

#### 2.2.1 Standardised wheelchair mobility tests

**Figure 1:**
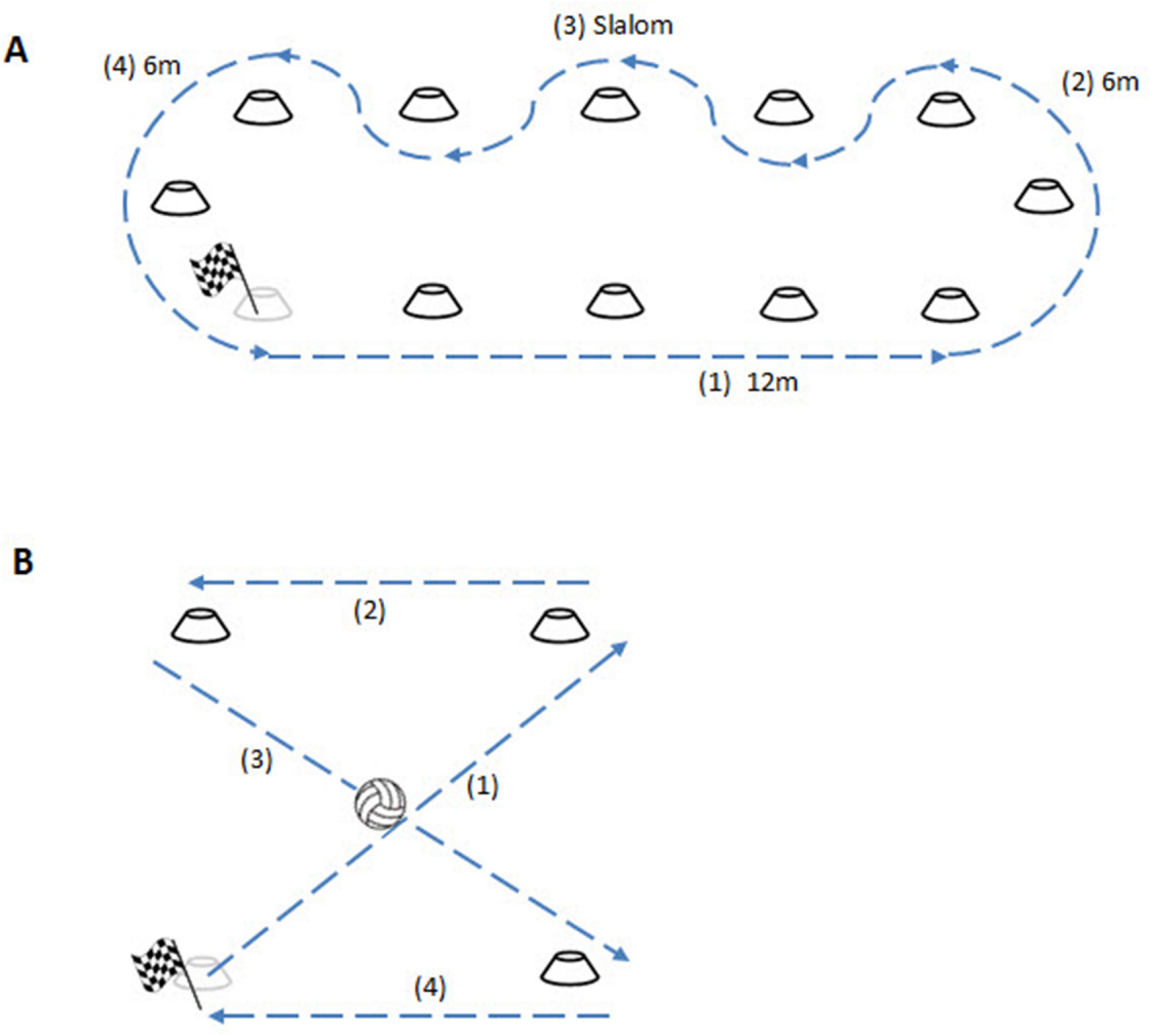
Set-up of Sprint-curve-slalom-curve and X-test without and with ball. This figure shows the setup for **(A)** the Sprint-curve-slalom-curve and **(B)** the X-test without and with ball.

For the standardised on-court wheelchair mobility performance tests, a selection of WR related tests was made from the protocol for wheelchair basketball as described by de Witte et al. (16), with the addition of a 20 m sprint and an X-test without the ball:

1. 20-m sprint
2. 12-m sprint with full stop
3. Intermittent sprint: 3-meter sprint, stop, 3-meter sprint, stop, 6-m sprint with full stop
4. Sprint-curve-slalom-curve (see Figure 1A)
5. Turn on the spot 180°, once to the right and once to the left.
6. Turn on the spot 90°, stop, turn 90° in the same direction, once to the right and once to the left
7. X-test, without the ball (see Figure 1B)

All tests were performed in this sequence, with a minimum of 10 s in-between tests. The 20 m sprint was added since it was assumed that in WR, long sprints are more common than in wheelchair basketball, where sprints typically do not exceed the 12 m (17). All these tests were performed once without a practise trial. The x-test without the ball was added to test for the combined skill of acceleration and manoeuvrability. This test was performed twice. Before the first test, a practice run was performed, because the x-test was a rather complex test that is no part of a routine practise drill. For x-test a square of 4 cones, 4 m apart was used (see Figure 1B). The athletes were asked to start at the lower left corner and push their wheelchair in a x-figure around the cones (diagonally, straight, diagonally, straight). The average outcome time was used for analysis.

During all wheelchair tests, wheelchair mobility performance was measured using two inertial sensors, one placed on the right wheel and one on the camber bar. It was sometimes not possible to place the sensor on the camber bar because the seat touched the camber bar. In such cases, the sensor was mounted on a nearby part of the frame, maintaining the same orientation. This placement did not affect the measurements, as only the horizontal rotational velocity was used, which remains consistent across all parts of the frame (18, 19).

Different sensors were used due to advancements in sensor technology during the study period. For the 2022 Wheelchair Rugby World Championship, Movesense HR + sensors (Vantaa, Finland) were used. In subsequent measurements, xIMU3 sensors (Bristol, UK) were employed (both with a sample frequency of 100 Hz). While both sensors provide equivalent signal quality, the xIMU3 sensors offered enhanced usability by connecting via Wi-Fi to a single router or computer, simplifying data collection. In contrast, the Movesense sensors required individual Bluetooth connections to separate mobile devices for each athlete.

A custom-built Python (v3.11) script was used to process the inertial sensor data into wheelchair mobility performance outcomes per test (18–20). From the range of available outcomes, the maximal speed [m/s], maximal rotational speed [°/s], and mean absolute rotational acceleration [°/s^2^] were selected for further analysis.

#### 2.2.2 Standardised ball tests

The standardised ball test set consisted of three different tests: maximal throwing distance, precision throwing and the x-test with the ball (14). Because these tests were new, test-retest reliability was assessed. Data can be found in the Supplementary Material. Test-retest reliability was sufficient (*r* > 0.6) for all tests, except for the one-handed precision throw at 25% and the two-handed precision throw at 75%. The *maximal throwing distance test* was executed one-handed (one-handed throw) and two-handed (two-handed throw), and for each condition participants had three attempts to throw as far as possible. Maximal throwing distance was visually assessed perpendicular to a tape measure that was taped to the floor. The largest distance was used for analysis and to determine the distance for the precision throwing tests. The *precision throwing test* was also performed one- and two-handed, at 25% and 75% of the maximal distance thrown during the maximal throwing distance test. The percentage of 75% for the long distance throw was chosen so that despite a variation in the maximum throwing distance between attempts, the long distance precision throw could be achieved in all attempts. The 25% distance for the short distance precision throw was chosen to have a meaningful distance, even for the athletes with a shortest maximum throwing distance (<3 m). Participants had three attempts to hit a round target (Ø 30 cm) that was taped to the wall with the centre at a height of 1 m. This was approximately at chest height for all athletes. There were no standardised rest times between attempts for distance and precision throwing. The participant started each throw after he indicated he was ready. Each attempt was videotaped from behind the throwing shoulder of the participant and later on the distance from the target on the wall to the ball was calculated using custom video analysis software in Matlab and a reference distance that was also taped on the wall. The Matlab mm player (Robert Walter, Robert Schleicher, 2009) was used to retrieve the pixel coordinates of the centre of the ball when it hit the wall, the centre of the target, and 3 reference points on the wall. Based on the pixel locations and the known distance between the reference points the distance between the centre of the ball and the target was calculated. The average distance to the centre of the target (in cm) was used as the outcome measure. For the x-test with the ball, the same circuit as in the x-test in wheelchair mobility was used. But in this test, the ball was picked up from the floor at the first (diagonal) line, and each next line the athletes had to dribble two times (so 6 dribbles in total). The total time to complete the x-test with the ball was recorded and the time in s of the x-test without the ball was subtracted to calculate an outcome measure for time (s) needed for ball pick-up and dribbling. Each athlete had two attempts and the average time was used for analysis.

### 2.3 Data analysis

First, a descriptive visual analysis was performed. All individual outcome measures (*Y*-axis) were plotted with the sports class of the athletes on the *X*-axis and a distinction between athletes with CI and athletes with other impairments (OI). Median values and 95% confidence intervals (CI) were calculated for all outcome measures. Spearman's Rank correlation was assessed for athletes with CI and OI for each outcome measure. Correlations of 0.6 and more were considered strong correlations (21). Lastly, it was calculated if there were any differences between participants with CI and OI using a Mann-Whitney U test. Both the Figures for the descriptive analysis were made and the correlations were calculated using Matlab [MATLAB version: 9.13.0 (R2022b), Natick, Massachusetts: The MathWorks Inc.; 2022]. The test-retest reliability for the ball tests was assessed using SPSS (IBM Corp. Released 2023. IBM SPSS Statistics for Windows, Version 29.0.2.0 Armonk, NY: IBM Corp).

## 3 Results

The median NRS score prior to the tests was 4 (range 1–7) and after the test was also 4 (range 1–9). In 35 athletes, the NRS score stayed the same. In 13 athletes there was an increase in fatigue after the tests. In 10 athletes, there was a decrease of fatigue after the tests. There were 10 athletes with NRS score >5 before or after the tests, 6 of them had impaired muscle strength caused by spinal cord injury, 3 had CP and 1 had range impairment.

### 3.1 Standardised wheelchair mobility tests

**Figure 2:**
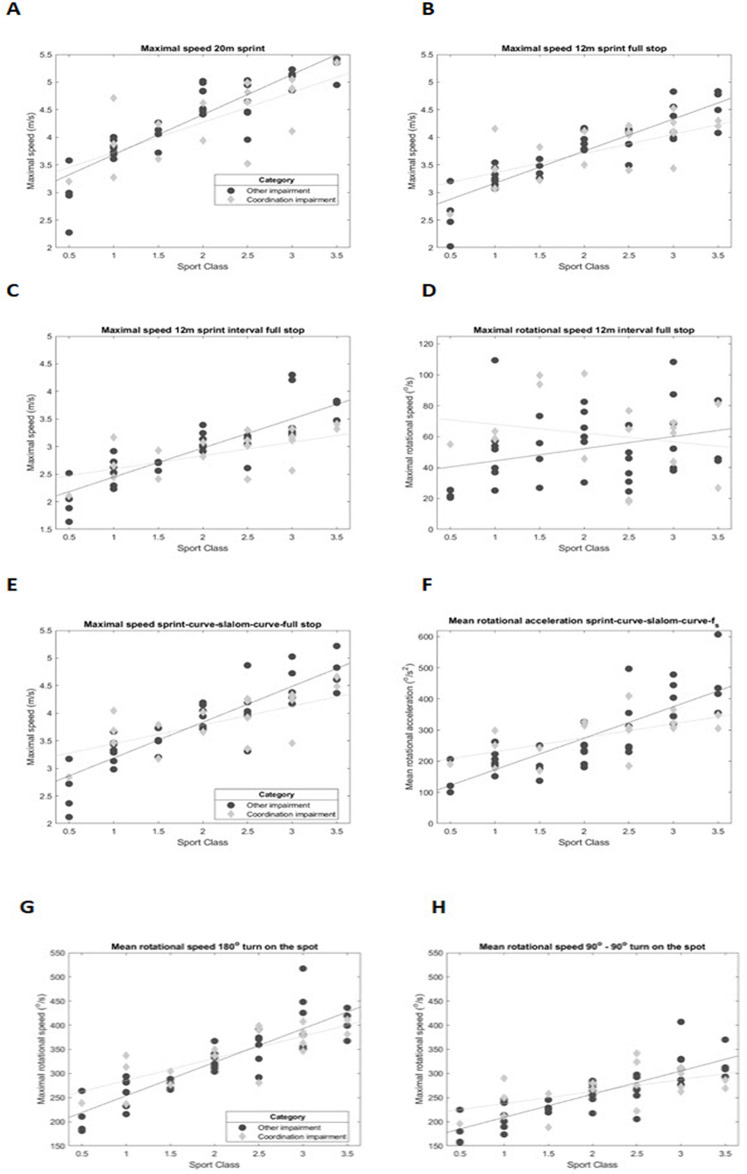
Results standardised wheelchair mobility tests. **(A)** Maximal forward speed in the 20 m sprint, **(B)** Maximal forward speed in the 12 m sprint with full stop, **(C)** Maximal forward speed in the 12 m interval sprint full stop, **(D)** Maximal rotational speed in the 12 m interval sprint full stop, **(E)** Maximal forward speed in the sprint-curve-slalom-curve full stop, **(F)** Mean rotational acceleration in the sprint-curve-slalom-curve full stop, **(G)** Mean rotational speed in the 180° turn on the spot, (H) Mean rotational in the 90°-90° turn on the spot.

The maximal forward and rotational speed in the straight sprints of 20 m, 12 m and 12 m interval, is shown in respectively Figures 2A–C for all athletes. In the 20 m sprint there is an increase of maximal speed with an increase in sports class, this is similar for athletes with CI and athletes with OI. Compared to the 20 m, the 12 m sprint is more dominated by acceleration and the ability to brake to a full stop at 12 m. The average maximal speed is similar for both impairment groups (3.78 ± 0.6 m/s for OI and 3.76 ± 0.56 m/s for CI), but one athlete with CI in sports class 1.0 is exceptionally fast in the 20 m sprint and two athletes in sports class 1.0 and 1.5 are exceptionally fast in the 12 m sprint with full stop. On the other hand, two athletes with CI sports classes 2.0 and 2.5 in the 20 m sprint and two athletes in the 2.5 and 3.0 sports class in the 12 m sprint with full stop are exceptionally slow.

The 12 m interval sprint shows a similar trend, but at a lower maximal speed compared to the 20 m and 12 m sprint for both groups. Two athletes in the 2.0 and 3.0 sports class are exceptionally slow in the 12 m interval sprint.

The maximal rotational speed in this interval sprint (Figure 2D) is determined by how well athletes can brake evenly, to maintain straight forward motion. In this graph there is a negative trend for the athletes with CI, with less rotational speed for athletes in the higher sports classes. Whereas no such trend is seen in athletes with OI.

The agility of the athlete is tested in the 12 m sprint- 6 m curve – slalom – 6 m curve with full stop (Figures 2E,F). The mean absolute rotational acceleration is a measure for how fast the athletes are able to change direction, from sprint to curve and in the slalom. Figure 2E shows the maximal forward speed with similar trends as the straight sprints with faster exceptions in the low point sports classes and slower exceptions in the high point sports classes in athletes with CI. The slower exceptions in the high point classes seem more pronounced in the mean rotational acceleration than in the maximal speed.

During the180° turn on the spot (Figure 2G) athletes can anticipate the expected turn, by already placing their hands in the optimal position on the rims. The average maximal rotational speed is similar in both groups, with the athletes with CI, again, showing some exceptionally fast athletes in the low point sports classes. In the 90°–90° turn on the spot (Figure 2H), repositioning of the hands is required, because this is an intermittent turn with a stop in the middle. In athletes with CI both fast exceptions in the low point sports classes and slow exceptions in the high point sports classes occur.

In the X-test without the ball, the time to complete the circuit is similar for athletes with and without. (Figure 4A).

### 3.2 Standardised ball tests

**Figure 3:**
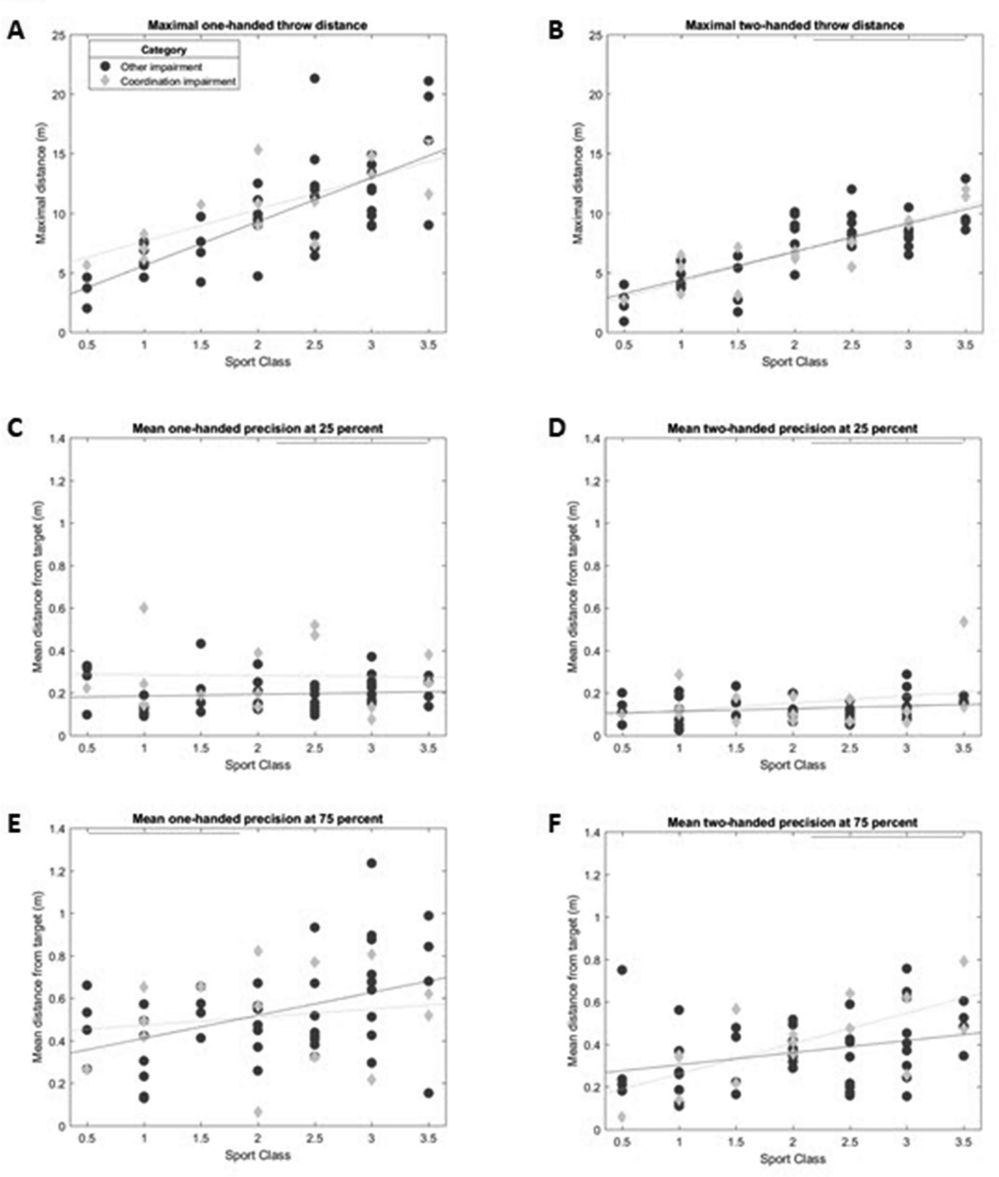
Results standardised ball tests. Maximal throwing distance (m) for **(A)** the one-handed throw and **(B)** the two-handed throw. Precision in m from the centre of the target (m) for **(C)** the one handed throw short distance, **(D)** the two handed throw short distance, **(E)** the one handed throw long distance, **(F)** the two handed throw long distance.

Figures 3A,B show the results for *maximal throwing distance*. All participants threw further with the one-handed throw compared to the two-handed throw, except for 5 athletes with OI, who threw approximately the same distance. In addition, sports class is correlated to maximal throwing distance for both the one-handed throw and the two-handed throw. There are no differences between athletes with CI and OI with regards to maximal throwing distance. Although two athletes with CI in sports classes 1.5 and 2.0 throw exceptionally far with the one-handed throw.

Figures 3C–F show the results of the *precision throwing test*. As expected, the throwing precision was better at 25% than at 75% of the maximal distance. In addition, throwing precision was better with the two-handed throw compared to the one-handed throw. In most conditions the throwing precision seems to be unrelated to sports class. However, at 75% of the maximal throwing distance, the distance to the target appears a little further for the high point sports classes. At 25% of the maximal throwing distance the group with CI shows more variation in throwing precision, especially at the one-handed throw, and most of them are less precise.

**Figure 4:**
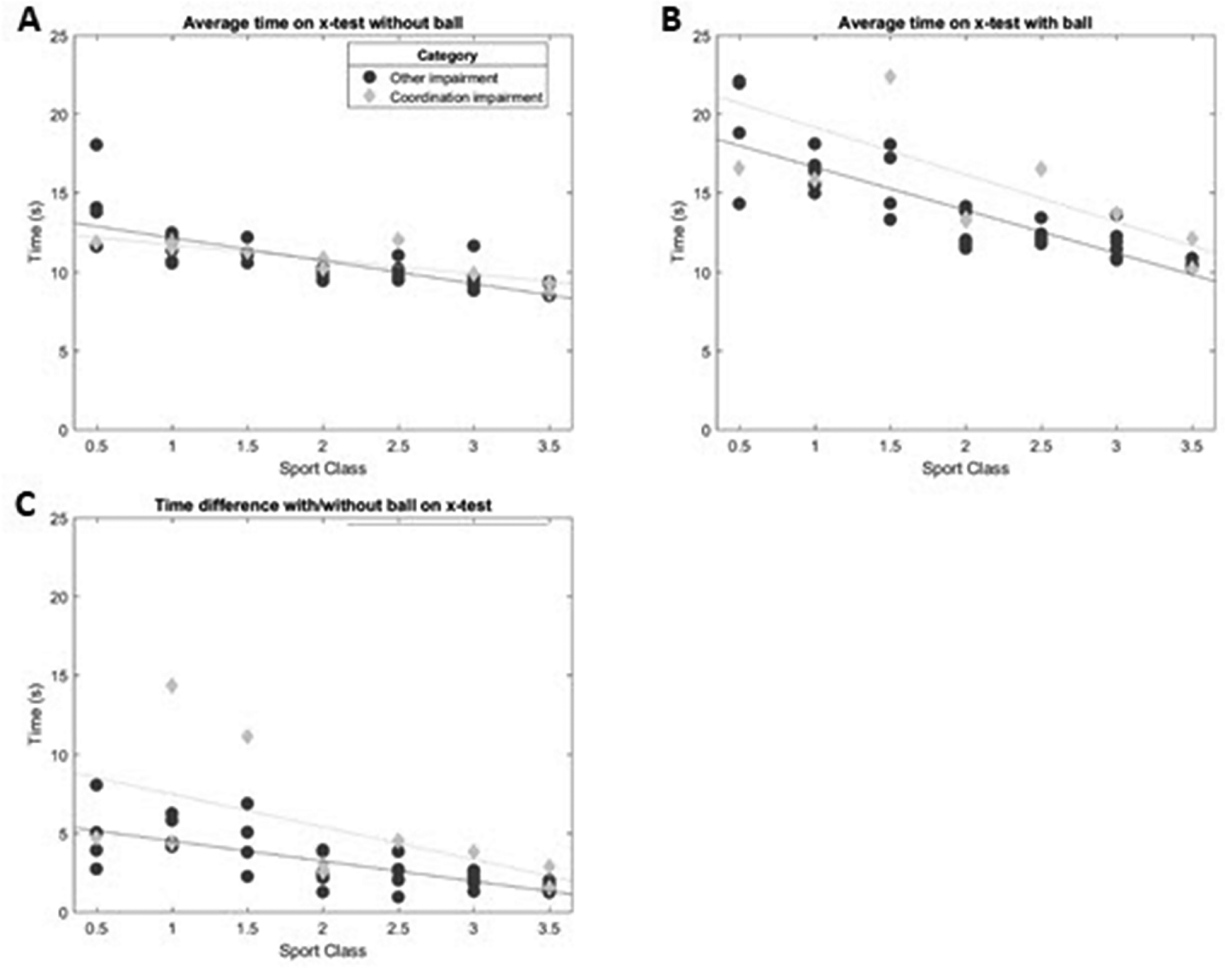
Results X test without and with ball. **(A)** average time X-test without ball, **(B)** average time X-test with ball and **(C)** time difference between X-test with and without ball.

Figure 4 shows the duration of the *X-test* with (4 B) and without ball (4 A) and the time difference between both tests (4 C). Overall, the X-test with ball takes longer than the X-test without the ball. In addition, athletes with a higher sports class are faster, both with and without ball. The duration of the X-test without ball is comparable between athletes with CI and OI. However, most athletes with CI were slower than athletes with OI when performing the X-test with the ball. The difference between the time on the X-test with and without the ball is larger in most athletes with CI, especially in athletes within the low point sports classes.

### 3.3 Correlation with sports class

The plots in the previous figures show a clear relationship for most outcomes with sports class. The strength of that relationship is expressed in the correlations as shown in the Spearman's rho values in Table 2. There is a significant and strong correlation between sports classes and all wheelchair mobility performance tests both for athletes with CI and OI athletes, except for all athletes in maximum rotational speed in the intermittent sprint, and athletes with CI in mean rotational acceleration in the Sprint-curve- slalom test and turn 90–90°. For the ball tests there was a significant and strong correlation between the maximal one-handed and the two-handed throw and the X-test with the ball in all athletes and for the long-distance two-handed precision throw in athletes with CI.

**Table 2:** The Spearman's rho correlations with classification split by impairment group, other impairment (OI) and coordination impairment (CI).

	Outcome measure	Median OI	95% CI OI	Spearman's rho with sports class OI	Median CI	95% CI CI	Spearman's rho with sports class CI	*P*-value difference OI and CI (Mann-Whitney U)
Wheelchair tests								
Sprint 20 m	Max speed (m/s)	4.50	4.1–4.6	**0**.**89**	4.43	3.9–4.9	**0**.**71**	0.69
Sprint 12 m	Max speed (m/s)	3.88	3.5–3.9	**0**.**86**	4.08	3.5–4.1	**0**.**71**	0.49
Intermittent sprint	Max speed (m/s)	3.02	2.8–3.1	**0**.**88**	3.07	2.7–3.2	**0**.**75**	0.79
	Max rot. speed (⁰/s)	52	45–59		63	49–83		0.11
Sprint-curve-slalom	Max speed (m/s)	3.95	3.6–4.0	**0**.**90**	4.02	3.6–4.2	**0**.**75**	0.62
	Mean rot. Acceleration (⁰/s^2^)	250	237–300	**0**.**85**	305	231–325	0.58	0.86
Turn on spot 180 R	Max rot. speed (⁰/s)	328	301–348	**0**.**86**	352	306–379	**0**.**81**	0.34
Turn on spot 180 L	Max rot. speed (⁰/s)	340	305–354	**0**.**92**	341	288–360	**0**.**72**	0.62
Turn 90–90 R	Max rot. speed (⁰/s)	267	243–278	**0**.**85**	265	238–288		0.81
Turn 90–90 L	Max rot. speed (⁰/s)	262	242–278	**0**.**86**	261	229–280	**0**.**60**	0.79
Time X-test	Mean time (s)	10.1	10.1–11.2	**−0**.**84**	10.8	9.7–11.5	**−0**.**73**	0.62
Ball tests								
One-handed	Max. distance (m)	9.0	8.1–10.9	**0**.**78**	10.8	8.9–12.9	**0**.**75**	0.19
Two-handed	Max. distance (m)	7.75	6.0–7.8	**0**.**73**	6.6	5.6–8.6	**0**.**80**	0.96
One handed 25%	Mean distance to target (m)	0.18	0.17–0.22		0.24	0.18–0.39		0.11
Two-handed 25%	Mean distance to target (m)	0.12	0.11–0.14		0.12	0.08–0.23		0.81
One-handed 75%	Mean distance to target (m)	0.50	0.45–0.59	0.42	0.56	0.39–0.67		0.70
Two-handed 75%	Mean distance to target (m)	0.35	0.31–0.41	0.38	0.43	0.33–0.54	**0**.**63**	0.19
Time X-test with ball	Mean time (s)	12.3	12.8–14.8	**−0**.**86**	13.7	12.0–19.9	**−0**.**75**	0.24
Time X-test with ball and without ball	Difference in time (s)	2.6	2.6–3.7	**−0**.**78**	3.8	2.0–8.7	**−0**.**68**	0.10

Only significant (*P* < 0.05) correlations are shown, with correlations over 0.60 in bold. The bottom part shows the correlations for the ball throwing tests, whereas the top shows the wheelchair mobility performance (WMP) tests.

## 4 Discussion

### 4.1 Key findings

The aim of this research was to assess if athletes with coordination impairment (CI) in WR show similar activity limitations as athletes with other eligible impairment types (OI) within the same sports class. In general, in both standardised wheelchair and ball activities, athletes with CI performed within the ranges of the athletes with OI in the same sports class. And correlations between sports class and standardised wheelchair and ball activities were generally high, both for athletes with CI and OI. This means that despite the current lack of impairment tests for CI, there is no reason to exclude athletes with CI from WR with the current classification system. However, there is still a need for valid and reliable impairment tests for CI according to the principles of the IPC classification code to determine the impact of impairment on performance in athletes with CI (2–4). The research that is taking place (11) should be continued, and valid and reliable tests for CI should be implemented in classification as soon as they become available. To the best of our knowledge, there are no studies in wheelchair team sports that assess the relationship between coordination impairment and activity limitation or assessed the comparison between several eligible impairment types (22).

The need for specific classification of coordination impairment is different for each Paralympic sport, also depending on the competition format. Some individual sports, like athletics and boccia, have separate competitions for athletes with CI and athletes with OI (22, 23). For these sports, minimum impairment criteria and determining the number and the borderline of sports classes is sufficient (2–4). However, in wheelchair team sports in which athletes with different impairment types compete in one competition, such as wheelchair basketball, wheelchair handball and wheelchair rugby, this is not sufficient. In addition, a comparison between impairment types and the impact on the ability to perform activities that determine proficiency in each sport should also be assessed. Only if the impact of different impairment types on the ability to perform is similar, athletes with different impairment types can continue to compete in one class in one competition. The present study is the first study in which this comparison is explored.

In wheelchair mobility performance tests in which only linear velocity is measured in a 20 m and 12 m sprint, there is a strong correlation between sports class and maximal forward velocity with an increase in forward velocity with an increase in sports class, both for CI and OI. Within that same activity, athletes in the low point classes with CI show a higher rotational velocity compared to athletes with OI. On the other hand, they show lower rotational velocity in a turn on the spot in which they have to reposition their hands on the wheel (90°–90° turn). In a previous study in Boccia, it was found that grasping, releasing and precision in direction of movements in the sagittal plan were affected in athletes with CI. In the more impaired athletes this was more affected (25). Similarly, the differences in rotational velocity between athletes with CI and OI may be caused by a difficulty with timing and positioning the hands on the wheels or push rims in athletes with CI, to produce a well targeted, even or uneven push.

It is striking however, that in tests in which acceleration plays a more important role (12 m sprint full stop) the low point athletes (sports class 0.5–1.5) with CI show a few athletes who perform considerably better than all the other athletes in the same sports class. This may be because acceleration is mainly determined by trunk impairment. The less trunk impairment, the faster an athlete can accelerate (26). Athletes with OI in the low point classes usually have severe trunk impairment with complete paralysis of the trunk muscles caused by spinal cord injury, resulting in trunk score 0 (8). Athletes with CI have some retained strength (trunk scores ≥ 0.5) (27, 28). Some trunk muscle strength, despite trunk coordination impairment in the low point athletes with CI compared to athletes with OI is most likely the reason why some of them perform relatively well in the 12 m sprint. However, in athletes with CI both static and dynamic sitting balance are affected compared to persons without CI (27, 28). In the sports classes 2.0–3.5 in which many athletes with OI have some to normal trunk strength, acceleration seems to be affected similarly by trunk strength impairment and trunk coordination impairment. In addition to differences in acceleration, low point athletes with CI show higher angular velocity in agility tests (sprint-curve slalom) and in the 180° turn on the spot. This may be caused by differences in the ability to grasp the wheels or the push rims. In a study in wheelchair racing, grip strength showed a strong relationship with velocity, both in 0–15 m sprint and in top speed (29). Athletes with OI in the low point sports classes usually have no hand function and no functional grasp or release. But athletes with CI in the low point sports class can have hand function resulting in an active grasp and release, which may help them in holding the wheel of the push rim to facilitate a turn (6, 25).

In summary, athletes with CI seem to be able to compensate to some extent for the difficulty in aiming the position and the timing of their arms in wheeling by use of trunk and hand function. This results in similar performance in linear velocity compared to athletes with OI. But in low point athletes with CI there may be some “over-compensation” by trunk and hand function in acceleration and turning. Therefore, we advise the classifiers to take these activities into account in allocating the low point sports classes to athletes with CI.

With regards to ball activities, there is a strong correlation between sports class and maximal throwing distance, both one- and two-handed for CI and OI, with an increase in throwing distance with an increase in sports class. In the long distance (75%) throwing precision, there is a large variation in all athletes (CI and OI) that was seen both within one athlete in the limited test-retest reliability, and between athletes. This is due to the higher velocity of the throwing movement, which results in less precision and thus in a higher variation (31). Due to this large variation between individual athletes, differences between athletes with CI and OI were not found. However, precision in a short distance, low velocity throwing movement is generally high (31). High precision was also found in short distance two-handed throwing in athletes with CI and OI and in one-handed short distance throwing in athletes with OI. But less so in athletes with CI. Opposite to wheeling and two-handed throwing, which are partially closed chain movements, one-handed throwing is an open kinematic chain movement (31). Open kinematic chain movements require more complex motor planning and are therefore more impacted by CI than closed chain kinematic movements. This likely explains why a difference in throwing precision between athletes with CI and OI was only found in the one-handed throw over the short distance.

Opposite to the throwing tasks, which were single tasks in which the timing of the action could be chosen by the athlete, the X-test with the ball required multitasking in which the pace and the timing of the ball handling was partially determined by the wheeling. All athletes, regardless of their impairment type, were slower in the X-test with the ball compared to the X-test without the ball. But in the athletes with CI the difference was larger than in the athletes with OI. Moreover, the difference was larger in low point athletes with CI than in high point athletes with CI. This indicates a major impact of CI on ball handling in a combined task. In summary, athletes with CI show more impact of their impairments on the ball activities one-handed short distance throw and ball handling in a combined task with wheeling than athletes with OI in the same sports class.

### 4.2 Study strengths

The present study is the first study on athletes with CI in wheelchair team sports. Although persons with CI form a potential large group of athletes, because it is the most prevalent impairment at a young age (32), their numbers are still low in wheelchair team sports. One reason may be the lack of understanding of the impact of their impairment on activities in these sports by teammates, coaches and classifiers. With the findings of this study, their participation can hopefully be enhanced. Equipment, training and skill development can also impact activities in WR and as such be confounders in research in which the impact of impairment on activities is assessed. However, by measuring world elite athletes, equipment, skills and training will be optimised and any differences between athletes will probably not be caused by these potentially confounding factors. Finally, the present study is the first study that includes standardised, objective measure for ball activities, based on the existing literature (14). Based on the study findings, the tests for ball activities have acceptable test-retest reliability and are feasible for future research and classification. An important impact of CI on these ball activities has been seen in this research. And this impact is different from the impact of OI. This underpins the inclusion of ball activities both in activity observation in classification and in research on CI.

### 4.3 Study limitations

The absolute number of athletes with CI in this study was low. But compared to the number of athletes with CI that are worldwide registered to have ever played WR (5% of all athletes) since the start of the sport, it is high (8). The researchers put in every effort to enrich the study population of athletes with CI to 25%, with a representation of athletes with CI in all sports classes. It needs to be noted that the results of the statistical comparison between OI and CI highly depends on the sports class of the athletes in each group. As the median sports class is 2.0 for both groups, we think this justifies the statistical comparison between groups, nevertheless the results should be interpreted with care. No objective tests for impairment were done in the present study, because they are not yet available. Athletes with CI had been allocated a sports class by well trained, international classifiers. But this sports class was based on expert opinion and the observation of non- standardised activities. This means that we cannot detect any relationship between objective measures of CI and activity limitation. We can only support that the activity limitation that is observed in a non-standardised way in athletes with CI by classifiers, is largely similar to the activity limitation in athletes with OI in the same class who had been classified by a (partially) evidence-based classification system including objective impairment tests (2–4, 6–9).

Another potential limitation of the study is the use of two different types of inertial measurement units (IMUs) during data collection. However, this does not affect the quality of the signal, as both Movesense HR + and xIMU3 sensors provide equivalent data accuracy for the selected outcomes. The difference between the two sensor types lies primarily in usability. The xIMU3 sensors transmit data via Wi-Fi and can also store data locally on the sensor, offering greater flexibility and efficiency during data collection. In contrast, the Movesense sensors rely on Bluetooth and require a separate mobile device for each set of sensors, which is more logistically demanding. Despite these differences, the consistency of the data processing pipeline ensured comparable results across all measurements.

Finally, fatigue can play a role in test performance with a decline of the performance at an increase of fatigue. And this may affect athletes with CI in a different way from athletes with OI. Therefore, NRS scores for fatigue were asked directly prior to the tests and after the tests. The NRS fatigue scores were low (median 4) and did not increase in the majority (15) of the athletes. NRS scores that were considered high were evenly distributed amongst impairment types. Because of the low values, the lack of an increase, and the even distribution over the participants with CI and OI, it is unlikely that fatigue has confounded the results.

### 4.4 Future perspectives

Based on the present findings we can already improve the classification of athletes with CI in WR to some extent. Standardised activity tests, with objective outcome measures and sufficient test-retest reliability, which are feasible for classification have been introduced. The impact of CI on performance in WR is most pronounced in wheelchair activities that require adequate timing and positioning of the hands on the wheels to result in well directed push and in combined wheelchair and ball activities and in one-handed short distance precision throwing. Furthermore, athletes with severe CI, but some trunk muscle strength and or grasp and release function in their hands may be able to compensate to some extent for their CI in wheelchair manoeuvring, but not in ball activities. This can be taken into account in observation of activities allocating the athlete sports class by experts.

With regards to future research, assessing the relationship between impairment tests for CI and activity limitation (3), short distance one-handed precision throws and combined chair and ball activities should be included in the research protocol. Whereas for wheelchair activities, the research protocol can be limited to intermittent sprint, sprint-curve-slalom-curve, and turn on the spot 90°–90°.

## Data Availability

The raw data supporting the conclusions of this article will be made available by the authors, without undue reservation.
