# Supplementary material for: Standardised activities in wheelchair rugby, comparison between athletes with coordination impairment and athletes with other impairments
Source: Front Sports Act Living. 2025 Jan 14;6:1519232. doi: 10.3389/fspor.2024.1519232 (PMC11772419; doi:10.3389/fspor.2024.1519232)
Supplement: Supplementary file 1 [file Datasheet1.docx]

Supplement 1: Test-retest reliability of ball handling activities

| **Ball tests** | **N** | **Mean (SD) test** | **Mean (SD) Retest** | **ICC* (p-value)** |
| --- | --- | --- | --- | --- |
| Max. distance one-handed (m) | 12 | 9.1 (4.2) | 9.3 (3.9) | **0.96 (<0.001)** |
| Max. distance two-handed (m) | 12 | 5.9 (2.9) | 5.6 (2.3) | **0.86 (<0.001)** |
| Mean one-handed precision at 25% (m) | 12 | 0.24 (0.10) | 0.24 (0.11) | -0.78 (0.994) |
| Mean two-handed precision at 25% (m) | 12 | 0.15 (0.07) | 0.16 (0.06) | **0.58 (0.022)** |
| Mean one-handed precision at 75% (m) | 12 | 0.66 (0.24) | 0.65 (0.30) | **0.86 (<0.001)** |
| Mean two-handed precision at 75% (m) | 12 | 0.35 (0.18) | 0.37 (0.18) | 0.30 (0.175) |

**Intraclass correlation coefficient: two-way mixed model with absolute agreement.*
