# Supplementary material for: Standardised activities in wheelchair rugby, comparison between athletes with coordination impairment and athletes with other impairments
Source: Front Sports Act Living. 2025 Jan 14;6:1519232. doi: 10.3389/fspor.2024.1519232 (PMC11772419; doi:10.3389/fspor.2024.1519232)
Supplement: Supplementary file 2 [file Datasheet2.pdf]

Supplement 1: Test retest reliability of ball handling activities

Ball tests	N	Mean (SD) test	Mean (SD) Retest	ICC* (p-value)
Max. distance one-handed (m)	12	9.1 (4.2)	9.3 (3.9)	**0.96 (<0.001)**
Max. distance two-handed (m)	12	5.9 (2.9)	5.6 (2.3)	**0.86 (<0.001)**
Mean one-handed precision at 25% (m)	12	0.24 (0.10)	0.24 (0.11)	-0.78 (0.994)
Mean two-handed precision at 25% (m)	12	0.15 (0.07)	0.16 (0.06)	**0.58 (0.022)**
Mean one-handed precision at 75% (m)	12	0.66 (0.24)	0.65 (0.30)	**0.86 (<0.001)**
Mean two-handed precision at 75% (m)	12	0.35 (0.18)	0.37 (0.18)	0.30 (0.175)

**Intraclass correlation coefficient: two-way mixed model with absolute agreement.*

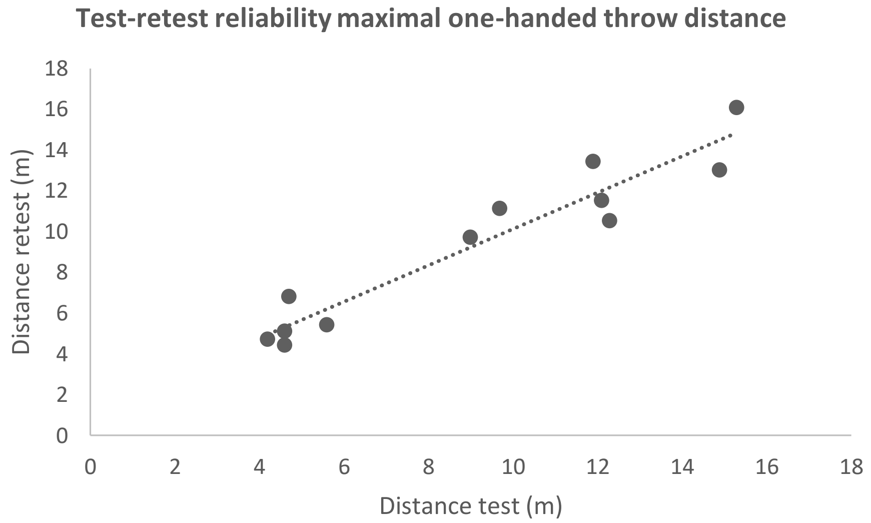

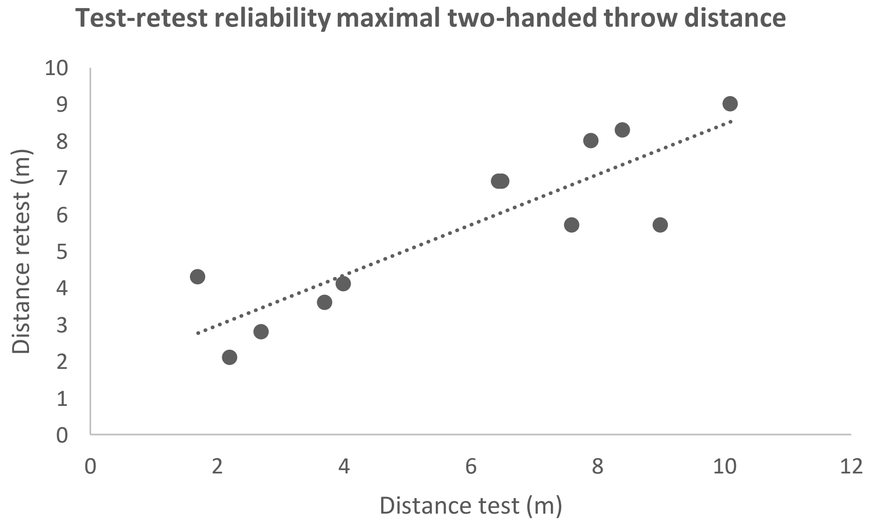

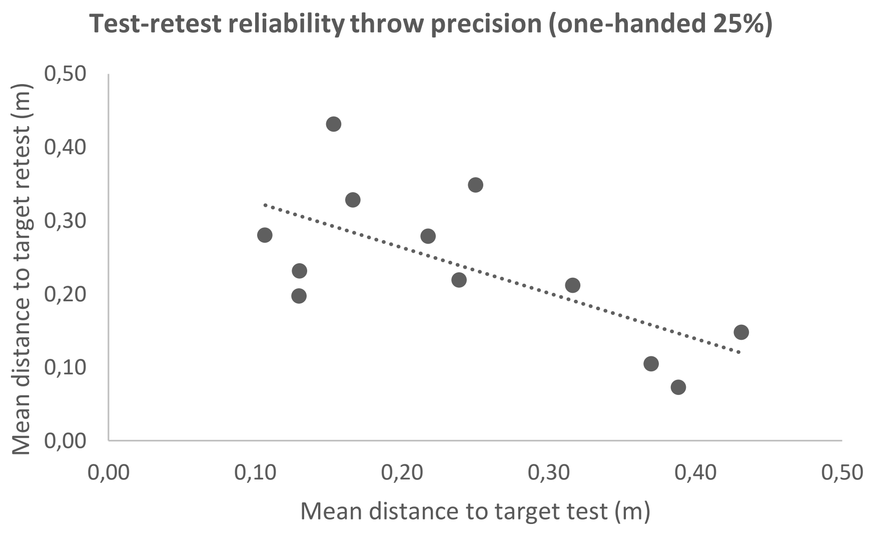

Test-retest reliability throw precision (one-handed 25%)
Mean distance to target retest (m)
0,50
0,40
0,30
0,20
0,10
0,00
0,00
0,10
0,20
0,30
0,40
0,50
Mean distance to target test (m)

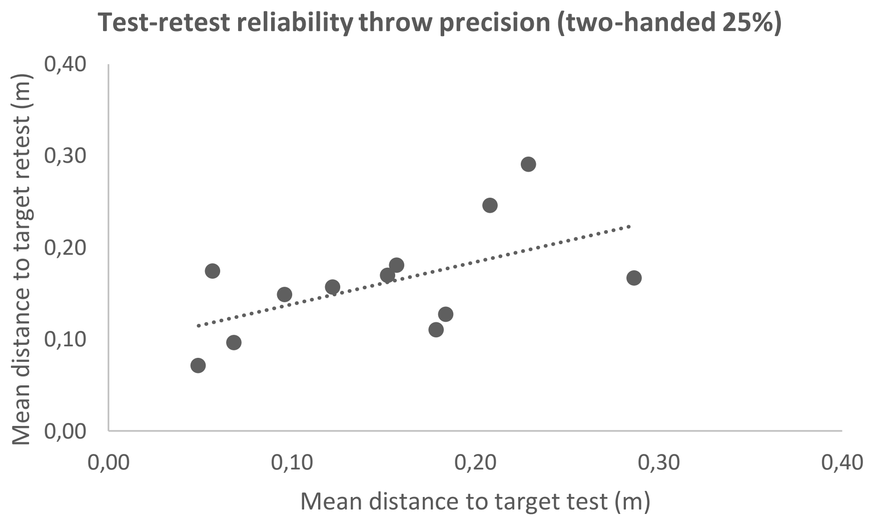

Test-retest reliability throw precision (two-handed 25%)
Mean distance to target retest (m)
0,40
0,30
0,20
0,10
0,00
0,00
0,10
0,20
0,30
0,40
Mean distance to target test (m)

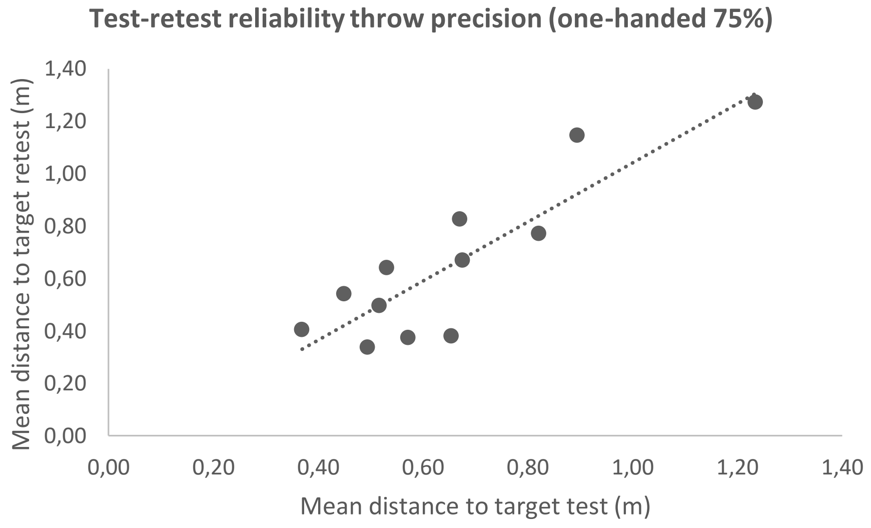
Test-retest reliability throw precision (one-handed 75%)
1,40
1,20
1,00
0,80
0,60
0,40
0,20
0,00
Mean distance to target retest (m)
0,00
0,20
0,40
0,60
0,80
1,00
1,20
1,40
Mean distance to target test (m)

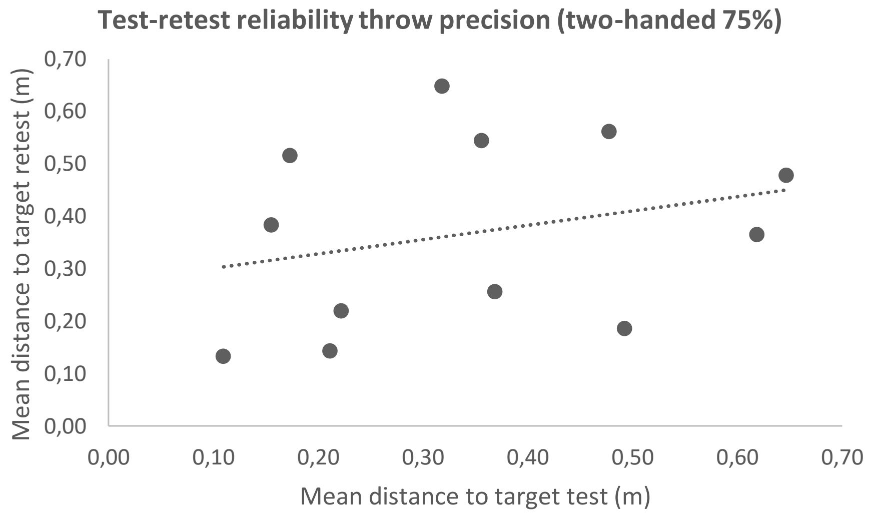
Test-retest reliability throw precision (two-handed 75%)
0,70
0,60
0,50
0,40
0,30
0,20
0,10
0,00
Mean distance to target retest (m)
0,00
0,10
0,20
0,30
0,40
0,50
0,60
0,70
Mean distance to target test (m)
